# *hGFAP*-mediated *GLI2* overexpression leads to early death and severe cerebellar malformations with rare tumor formation

**DOI:** 10.1016/j.isci.2023.107501

**Published:** 2023-07-28

**Authors:** Judith Niesen, Irm Hermans-Borgmeyer, Christina Krüger, Melanie Schoof, Franziska Modemann, Ulrich Schüller

**Affiliations:** 1 Mildred Scheel Cancer Career Centre HaTriCS4, University Medical Centre Hamburg-Eppendorf, 20251 Hamburg, Germany; 2 Research Institute Children’s Cancer Centre, 20251 Hamburg, Germany; 3 Department of Pediatric Hematology and Oncology, University Medical Centre Hamburg-Eppendorf, 20251 Hamburg, Germany; 4 Scientific Service Group for Transgenic Animals, University Medical Centre Hamburg-Eppendorf, 20251 Hamburg, Germany; 5 Department of Oncology, Hematology and Bone Marrow Transplantation with Section Pneumology, II. Department of Internal Medicine, University Medical Centre Hamburg-Eppendorf, 20251 Hamburg, Germany; 6 Institute of Neuropathology, University Medical Centre Hamburg-Eppendorf, 20251 Hamburg, Germany

**Keywords:** Biological sciences, Molecular biology, Cancer

## Abstract

The zinc-finger transcription factor *GLI2* is frequently amplified in childhood medulloblastoma of the Sonic-hedgehog type (SHH-MB), with or without amplification of *NMYC* or deletion of *TP53*. Despite the aggressive tumor behavior, tumorigenesis is not well understood, and adequate mouse models are lacking. Therefore, we generated mice with a *GLI2* overexpression under control of the *hGFAP*-promoter. These mice died within 150 days. The majority only survived until postnatal day 40. They displayed severe cerebellar hypoplasia, cortical malformations, but no brain tumors, except for one out of 23 animals with an undifferentiated hindbrain lesion. Additional loss of p53 did not result in cerebellar tumors, but partially rescued the cerebellar phenotype induced by *GLI2* overexpression. Similarly, the combination of *GLI2* and *NMYC* was neither sufficient for the development of SHH-MB. We therefore assume that the development of childhood SHH-MB in mice is either occurring in cellular origins outside the *hGFAP*-positive lineage or needs additional genetic drivers.

## Highlights

• Childhood SHH-medulloblastoma shows amplification of Gli2 and/or NMYC or deletion of TP53
• Mice with Gli2 overexpression show cerebellar malformations or rare brain tumors
• Further p53^Fl/Fl^ loss or NMYC^Fl/Fl^ expression affects cerebellar development and survival
• Additional alterations did not lead to MB

## Graphical abstract

**Figure:**
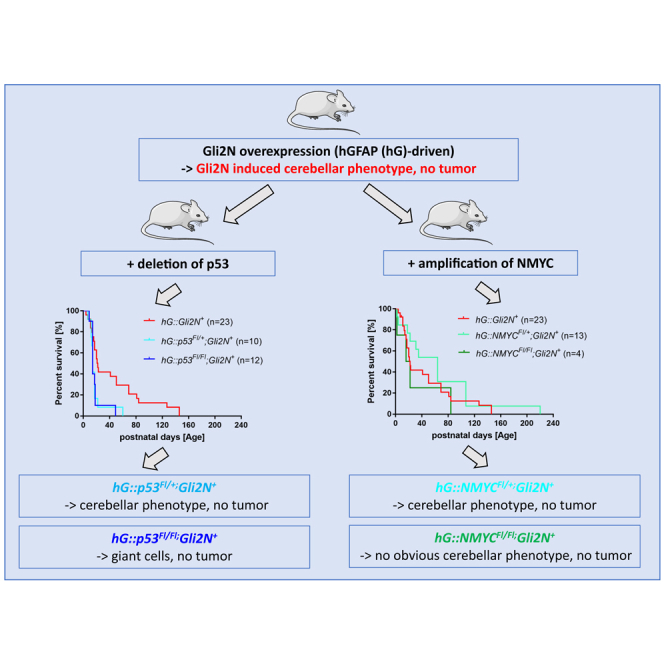

## Introduction

Sonic hedgehog (SHH) signaling is evolutionary conserved and essential for the development and differentiation of different organs, such as the central nervous system.^1^^,^^2^^,^^3^ The Shh signaling pathway comprises two transmembrane proteins Patched (PTCH), a 12-transmembrane protein, and Smoothened (SMO), a 7-transmembrane protein that acts as a signal transducer. PTCH binds Shh to activate the pathway, whereas, in the absence of ligands, PTCH interacts with and inhibits SMO.^4^ The hedgehog ligands disassemble PTCH inhibition of the transmembrane protein SMO. Additionally, SUFU (suppressor of fused homolog) acts as a negative intracellular regulator and segregates full-length Gli proteins in the cytoplasm. This all allows the transcriptional activation of *GLI* target genes.^1^^,^^5^ The GLI family consists of GLI1, GLI2, and GLI3.^5^^,^^6^ GLI1 is a downstream target of the SHH pathway. GLI2, also named GLI family zinc finger 2, and GLI3 are transcriptional mediators in SHH activation controlled by posttranscriptional and posttranslational alterations.^1^^,^^5^^,^^6^^,^^7^^,^^8^^,^^9^^,^^10^ *GLI2* also plays a key role as transcriptional effector in the SHH pathway, e.g., in the skin, and is known to be frequently amplified and overexpressed in pediatric brain cancer, such as pediatric SHH-medulloblastoma (MB). In SHH-MB, GLI2 occurs in combination with amplifications of *NMYC* or deletion of *TP53.*^6^^,^^10^^,^^11^ MB is the most common malignant brain tumor in children, representing 60% of childhood intracranial embryonal tumors and the leading cause of pediatric cancer deaths.^6^^,^^12^^,^^13^ While *PTCH1* mutations occur at all ages in SHH-MB, *SUFU* mutations are enriched in infant SHH-MB, and SHH-MB in adolescence/adulthood often harbors *SMO* mutations. However, the most aggressive subtype of SHH-MB occurs in childhood. Such tumors often appear histologically as severely anaplastic and are characterized by *TP53* mutations as well as *NMYC* and *GLI2* amplifications.^6^^,^^9^^,^^14^^,^^15^^,^^16^ *GLI2* is therefore believed to function as an important oncogene in SHH-MB.^17^^,^^18^^,^^19^ Regardless of all the research in MB, mechanisms and targetable components in childhood SHH-MB are still not fully understood. Intensive radiotherapy or chemotherapy that may lead to the cure of the disease often shows significant long-term side effects including cognitive deficits and the risk of secondary neoplasms.

Genetically engineered mouse models are a critical instrument to reveal cellular or molecular origins of SHH-MB and to perform preclinical testing of novel targeted therapies.^3^^,^^6^^,^^13^ High GLI2 expression has been detected in several transgenic MB mouse models,^20^^,^^21^^,^^22^ but it is not fully understood how GLI2 proceeds to drive cancer growth.^23^^,^^24^ In mice, constitutively active *GLI2* alone is not sufficient to induce MB, and it is tempting to speculate that additional mutational hits are needed for MB tumorigeneses.^7^ Conversely, the overexpression of mouse and human GLI2 under control of the bovine *K5* promoter (*K5-Gli2N*), which is active in keratinocytes of epidermal basal layer, leads to basal cell carcinomas of the skin.^10^^,^^24^^,^^25^^,^^26^ Similarly, another transgenic mouse model, which uses *GLI2A* (identical to *GLI2N*) expression in *Lgr5*^*+*^ stem cells, drives rapid gastric adenocarcinoma.^27^ *GLI2N* lacks an N-terminal repressor domain of *GLI2* and is much more active in GLI-responsive reporter and alkaline phosphatase activity assays than full-length human or mouse GLI2. Additionally, *GLI2N* produced basal cell carcinomas in transgenic mice, suggesting HH-independent cell proliferation.^10^^,^^28^^,^^29^ In human glial fibrillary acidic protein (*hGFAP*)*-cre::CLEG2*^*fl/+*^ mice that overexpress *GLI2N*, only very few animals develop tumors later in life in the cerebrum, spinal cord, or cerebellum. The *CLEG2* transgene includes a constitutively active *CAG* promoter, through which the expression of enhanced GFP (EGFP) is activated in absence of Cre expression. Additionally, a polyA sequence prevents the transcription of the *GLI2N* transgene flanked by *loxP* sites.^28^ However, the expression of constitutively active *GLI2* was not sufficient to reliably induce MB development.^28^ Whether or not *NMYC* and *TP53* mutations, which frequently occur in childhood MB together with *GLI2* amplifications, affect SHH signaling and particularly MB formation remains to be elucidated.

## Results

### Generation, breeding, and survival of genetically engineered mouse models

**Figure 1:**
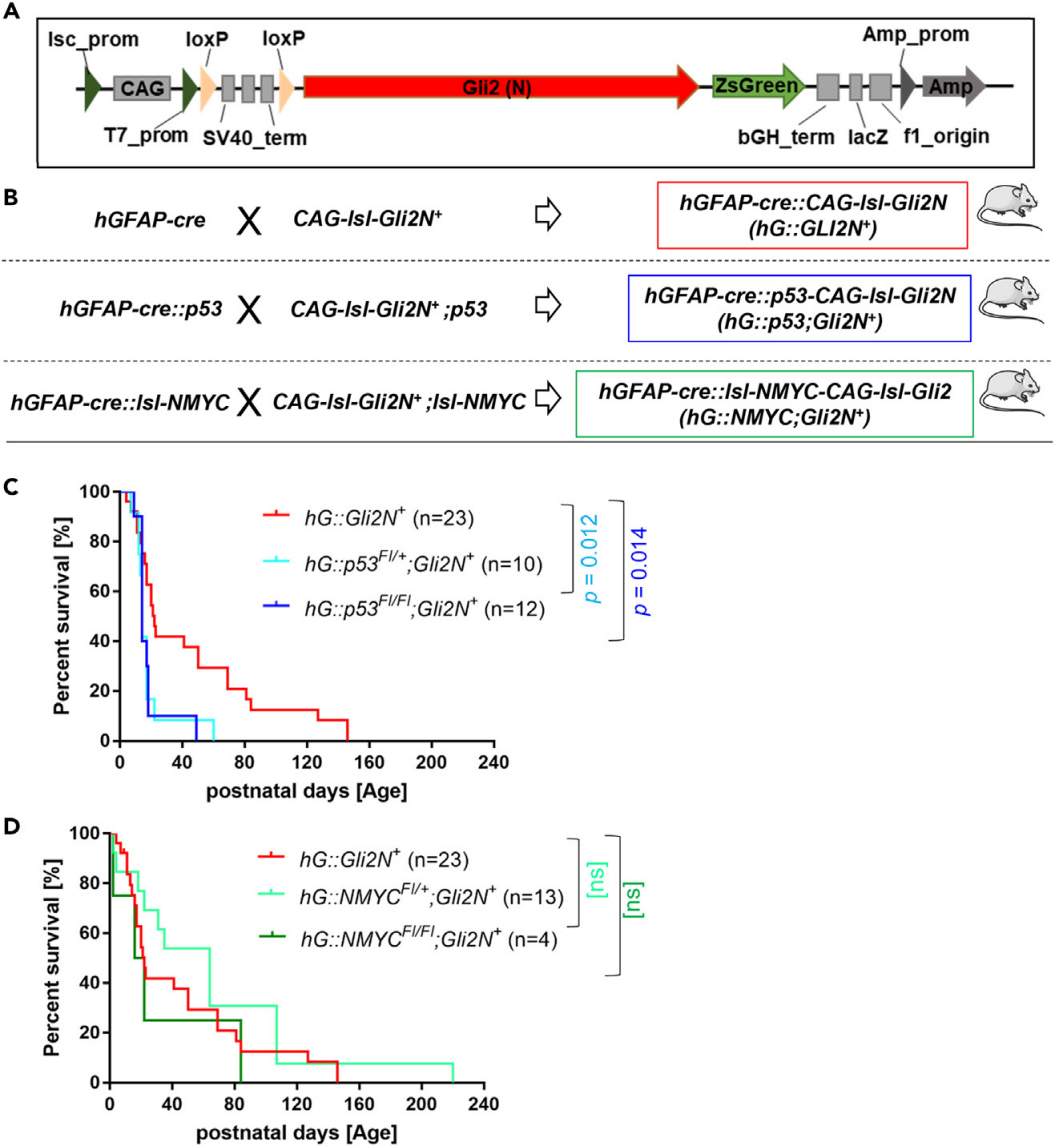
Vector plasmid construct, breeding schemes for Gli2 mice, and survival analysis (A) Illustrated is the vector plasmid construct, which was applied for pronuclear injection. Containing a lac-promoter part, the strong CAG-promoter (Cytomegalovirus early enhancer, the first intron of chicken beta-actin gene, the splice acceptor of the rabbit beta-globulin gene), T7 RNA-Polymerase promoter for transcription, the STOP cassette comprising three SV40-derived poly(A) signal repeats flanked by two *loxP* sites, the truncated GLI2N insert, a ZsGreen sequence (Zoanthus green fluorescent protein), a mammalian terminator bGH (bovine growth hormone), a lacZ gene coding for beta-galactosidase, f1_ori (DNA replication of bacteriophage f1 functions as a signal for initiation of viral strand synthesis and for its termination), and the *Amp*-promoter for ampicillin resistance. (B) The breeding schemes for the Gli2 mouse models are demonstrated. The transgenic line expressing Cre recombinase under the control of the human *glial fibrillary acidic protein* (*hGFAP*) promoter was crossed with *CAG-lsl-Gli2N* mice. The *CAG* promoter was applied to drive expression of a construct composed of floxed sites followed by an active form of *GLI2* lacking the N-terminal repressor domain (*GLI2N*), resulting in *hGFAP-cre::CAG-lsl-Gli2N* transgenic mice. To obtain double-mutant mouse models, *hGFAP-cre::p5*3 mice were intercrossed with *CAG-lsl-Gli2N*^*+*^*;p5*3 mice to achieve *hGFAP-cre::p53-CAG-lsl-Gli2N*^*+*^ (heterozygote or homozygote). *hGFAP-cre::lsl-NMYC* mice were crossed with *CAG-lsl-Gli2N;*^*+*^*lsl-NMYC* mice, resulting in *hGFAP-cre::lsl-NMYC-CAG-lsl-Gli2N*^*+*^*mice,* in heterozygous or homozygous results. More simplistic names were given to the mouse models as demonstrated in brackets. (C) Survival analysis of *hG::Gli2N*^*+*^ compared to *hG::p53*^*Fl/+*^*;Gli2N*^*+*^ (∗0.012 Log rank (Mantel-Cox) test); 95% CI of ratio 0.1967 to 1.056)) and *hG::p53*^*Fl/Fl*^*;Gli2N*^*+*^ (∗0.014 Log rank (Mantel-Cox) test); 95% CI of ratio 0.1774 to 1.126)). (D) In a second graph, the comparison of the survival of *hG::Gli2N*^*+*^ to *hG::NMYC*^*Fl/+*^*;Gli2N*^*+*^ (ns); 95% CI of ratio 0.2422 to 2.482 and *hG::NMYC*^*Fl/Fl*^*;Gli2N*^+^(ns); 95% CI of ratio 0.7242 to 2.662 is displayed.

To investigate the role of GLI2 in brain tumor development, we generated mice with a *GLI2* overexpression and additional *p53* deletion or *NMYC* overexpression. This combination is typical for the most aggressive types of SHH-MB, for which targeted therapies and appropriate models are lacking. The *GLI2N* gene, missing 328 N-terminal amino acids, was cloned into pCAG-loxPSTOPloxP-ZsGreen plasmid for pronuclear injection (Figure 1A). Resulting *CAG-lsl-Gli2N*^*+*^ mice were intercrossed with *hGFAP-cre::p53*^*Fl/Fl*^ and -*lsl-NMYC* mice in order to obtain *hGFAP-cre::p53;CAG::lsl-Gli2N*^*+*^ or *hGFAP-cre::lsl-NMYC;CAG::lsl-Gli2N*^*+*^ mice (Figure 1B). The median survival of *hGFAP-cre::CAG::lsl-Gli2N*^*+*^ (*hG::Gli2N*^*+*^) mice (n = 23) was 22 days, while the survival of *hGFAP-cre::p53*^*Fl/+*^*;CAG::lsl-Gli2N*^*+*^ *(hG::p53*^*Fl/+*^*;Gli2N*^*+*^*)* mice (n = 10) and *hGFAP-cre::p53*^*Fl/Fl*^*;CAG::lsl-Gli2N*^*+*^ (*hG::p53*^*Fl/Fl*^*;Gli2N*^*+*^*)* mice (n = 12) was only 14 days in both groups (Figure 1C). *hGFAP-cre::lsl-NMYC*^*Fl/+*^*;CAG::lsl-Gli2N*^*+*^ *(hG::lsl-NMYC*^*Fl/+*^*;Gli2N*^*+*^*)* mice (n = 13) and *hGFAP-cre::lsl-NMYC*^*Fl/Fl*^*;CAG::lsl-Gli2N*^*+*^ *(hG::NMYC*^*Fl/Fl*^*;Gli2N*^*+*^*)* mice (n = 4) showed a median survival of 64 and 19 days, respectively, which was not significantly different from the survival of the *hG::Gli2N*^*+*^ mice (Figure 1D). Besides the endpoint criteria, such as lower body weight or smaller than control littermates, neurological signs like imbalance or ataxia also counted and were probably a consequence from the dysfunction of the cerebellum.

### Overexpression of NMYC or deletion of p53 in combination with GLI2 overexpression is insufficient to drive cerebellar tumor formation

**Figure 2:**
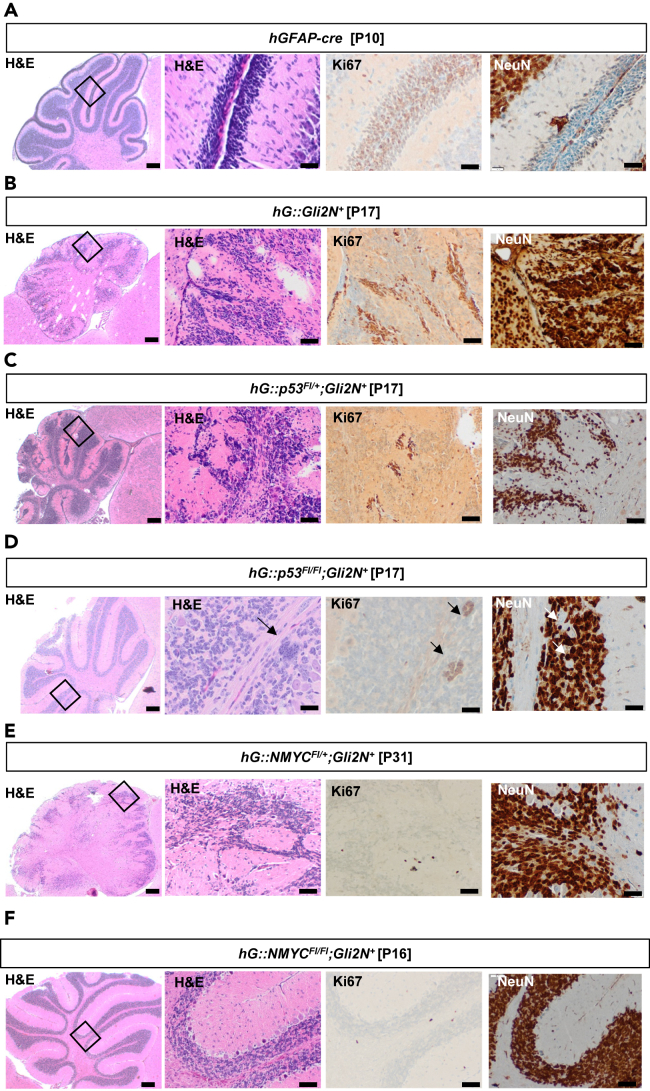
Brain sections after H&E, NeuN, and Ki67 staining Sagittal brain sections of paraffin-embedded tissues after H&E, Ki67, and NeuN staining. First columns display whole cerebellum (scale bar: 200 μm), second columns higher magnifications of the cerebellum (scale bar: 50 μm), and last columns the Ki67 staining (scale bar: 50 μm) and NeuN staining (scale bar: 50 μm). (A) *hGFAP-cre* control animal at postnatal day 10. (B) A representative *hG::Gli2N*^*+*^ mouse at postnatal day 17. Severe cerebellar disorder is demonstrated and proliferation with Ki67-positive staining as well as positive NeuN staining. (C) Heterozygous *hG::p53*^*Fl/+*^*;Gli2N*^*+*^ animal at postnatal day 17 with a similar disorder and proliferating cells within the cerebellar structure. (D) Homozygous *hG::p53*^*Fl/Fl*^*;Gli2N*^*+*^ animal at postnatal day 17 shows comparable cerebellum than the *hGFAP-cre* control mouse, but so-called giant cell structures both in the cerebellum and other parts of the brain (arrows). These giant cell structures are also proliferating (Ki67 staining) but did not show NeuN expression (arrows). (E) In 4 out of 13 *hG::NMYC*^*Fl/+*^*;Gli2N*^*+*^ mice, a cerebellar disorder was observed as exemplarily demonstrated here, but also tumor-like structures or no abnormalities in the whole brain at all. (F) Homozygous *hG::NMYC*^*Fl/Fl*^*;Gli2N*^*+*^ mice did not show any abnormalities in the brain structure at all.

Transgenic mice were examined for potential tumor development at different time points as soon as they had to be sacrificed due to symptoms. Representative cerebellar sections (H&E, Ki67, and NeuN staining) of 10- to 31-day-old mice of all generated genotypes are presented in Figure 2. Tumor formation was not observed. Figure 2A demonstrates a 10-day-old *hGFAP-cre* control mouse with regular structures of the cerebellum. In contrast, all of the 23 *hG::Gli2N*^*+*^ mice showed an unstructured cerebellum with ectopic cell clusters (Figure 2B). These mice were sacrificed due to signs, such as lower body weight, smaller than control littermates, strong ataxia, and imbalance, which was most likely caused by the cerebellar dysfunction. Likewise, *hG::p53*^*Fl/+*^*;Gli2N*^*+*^ mice (Figure 2C) showed cerebellar dysfunction. The clinical symptoms were comparable to those of *hG::Gli2N*^*+*^ mice. *hG::p53*^*Fl/Fl*^*;Gli2N*^*+*^ mice displayed a grossly normal structure of the cerebellum except for some multinucleated giant cells that were observed throughout the cerebella and show Ki67 staining, but no NeuN staining (Figure 2D, arrows). *hG::NMYC*^*Fl/+*^*;Gli2N*^*+*^ either showed a cerebellar disorder although proliferation was nearly absent at P31 as expected (Figure 2E) or a phenotype similar to *hG::NMYC*^*FlFl+*^*;Gli2N*^*+*^ mice. Two out of 13 *hG::NMYC*^*Fl/+*^*;Gli2N*^*+*^ mice did not show any phenotype. *hG::NMYC*^*Fl/Fl*^*;Gli2N*^*+*^ did neither show any cerebellar abnormalities (Figure 2F).

**Figure 3:**
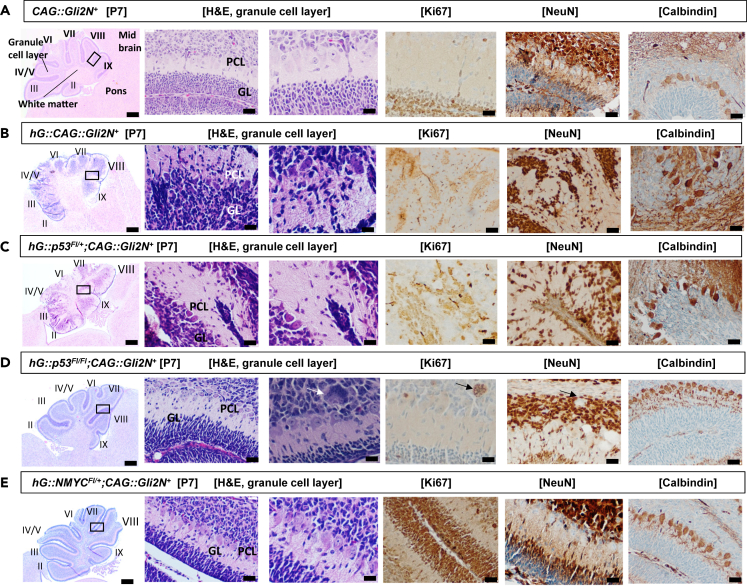
Brain sections of *CAG::Gli2N*^*+*^*, hG::Gli2N*^*+*^, *hG::p53*^*Fl/+*^*;Gli2N*^*+*^, *hG::p53*^*Fl/Fl*^*;Gli2N*^*+*^, and *hG::NMYC*^*Fl/+*^*;Gli2N*^*+*^*mice* at p7 after H&E, Ki67, NeuN, and Calbindin staining The mouse lines *CAG::Gli2N*^*+*^*, hG::Gli2N*^*+*^, *hG::p53*^*Fl/+*^*;Gli2N*^*+*^, *hG::p53*^*FlFl+*^*;Gli2N*^*+*^, and *hG::NMYC*^*Fl/+*^*;Gli2N*^*+*^ at p7 are demonstrated after H&E, Ki67, NeuN, and Calbindin staining. (A) CAG::Gli2N^+^ control mouse at p7 is demonstrated. The cerebellum and adjacent brain regions are labeled with their subunits in the first row (scale bar: 200 μm). In the H&E staining, the granule layer (GL) and Purkinje cell layer (PCL) are shown (scale bar: 50 μm) and in a higher magnification (scale bar: 20 μm) in the second and third row. Ki67, NeuN, and Calbindin staining is presented as specific staining (scale bar: 20 μm). (B) H&E, Ki67, NeuN, and Calbindin staining of *hG::Gli2N*^*+*^ mice. The cerebellum is unstructured and PCL and GL cannot be delineated as precisely as in the control. (C) Also in the demonstrated *hG::p53*^*Fl/+*^*;Gli2N*^*+*^ mouse, the cerebellum is unstructured, but shows Ki67, NeuN, and Calbindin staining of the PC as the *hG::Gli2N*^*+*^ mouse. (D) *hG::p53*^*Fl/Fl*^*;Gli2N*^*+*^ mice show giant cells in the H&E and Ki67 staining (white arrows) and the PCL and GL looks comparable to the one in the control (A). (E) H&E (scale bars: 200, 50, and 20 μM), Ki67 (20μM), and NeuN as well as Calbindin (20 μM) staining are demonstrated and are comparable to the control.

To gain insight into the postnatal stages of cerebellar development, we present in Figure 3 *Gli2N*^*+*^, *hG::Gli2N*^*+*^, *hG::p53*^*Fl/l*^*;Gli2N*^*+*^, *hG::p53*^*Fl/Fl*^*;Gli2N*^*+*^, and *hG::NMYC*^*Fl/+*^*;Gli2N*^*+*^ mice at postnatal day 7. The cerebellar cortex is arranged in three laminae: molecular layer, Purkinje cell layer (PCL), and granular layer (GL). In Figure 3 the Granule cell layer (GL) and Purkinje cell layer (PCL) are demonstrated. GL and PCL look characteristic for the control mouse (Figure 3A). For *hG::Gli2N*^*+*^ *and hG::p53*^*Fl/+*^*;Gli2N*^*+*^ mice in Figures 3B and 3C, the PCL and GL look unstructured. NeuN was predominantly found in the nucleus of post-mitotic granule neurons. It should be pointed out that giant cells found in *hG::p53*^*Fl/Fl*^*;Gli2N*^*+*^ mice were already visible at P7 and were positive for Ki67 (Figure 3D, arrow), while the remaining inner GL was negative. In contrast, NeuN expression was strong in the inner GL, but not in the giant cells itself (Figure 3D, arrows). For *hG::NMYC*^*Fl/+*^*;Gli2N*^*+*^ mice at p7, the Ki67 staining was strongly visible in the external GL, which was lost at p31 as seen in Figure 2E. Positive calbindin staining of the PCL was demonstrated in all mouse models (Figures 3A–3E, 6^th^ column, arrows).

### Additional features in subsets of hG:p53^Fl/Fl^;Gli2N^+^, hG::Gli2N^+^, and hG::NMYC^Fl/+^;Gli2N^+^ mice

**Figure 4:**
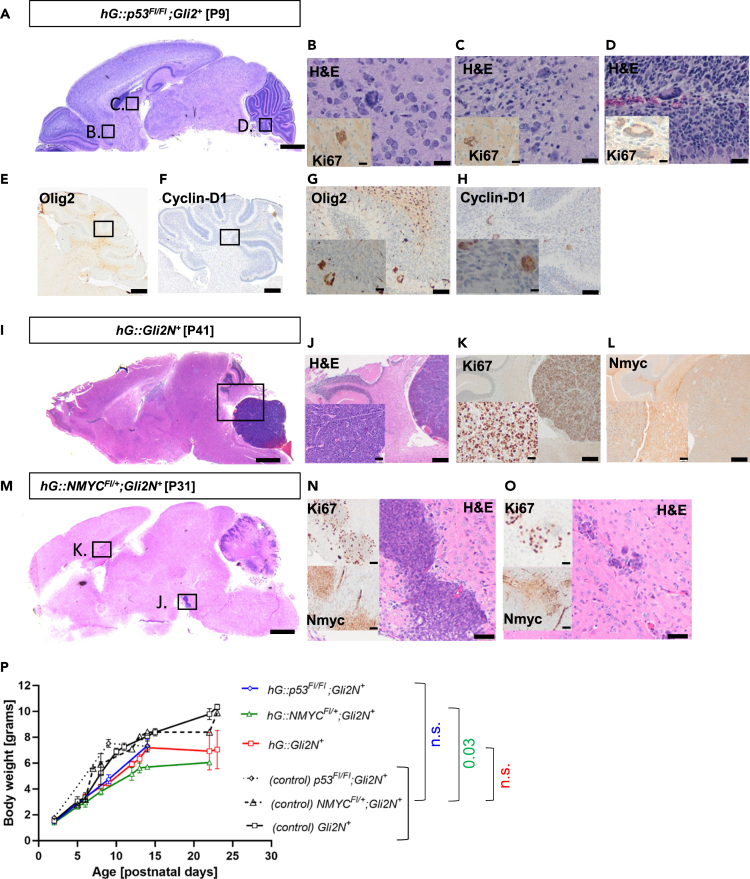
Representative images and staining of giant cells, tumors and tumor-like structures (A–H) Representative H&E and Ki67 stains of a *hG::p53*^*Fl/Fl*^*;Gli2N*^*+*^animal (Figures 4A–4D). Giant cells were found near the piriform cortex (Figure 4B), near the hippocampus area and caudate putamen (Figure 4C), and the cerebellum (Figure 4D). Scale bar is 1 mm for images of the whole brain demonstrated on the left, 20 μm for H&E in B, C, and D and 50 μm for Ki67 staining. E: *hG::p53*^*Fl/Fl*^*;Gli2N*^*+*^ mouse showed so-called giant cells, stained with Olig2 (Figure 4E, scale bar: 200μm) and Cyclin-D1 (Figure 4F, scale bar: 200 μm), both show expression. High-power images of Olig-2 expression giant cells (Figure 4G, scale bar: 20 and 50 μm) and Cyclin-D1 expressing giant cell (Figure 4H, scale bar: 20 and 50 μm) are demonstrated. (I–L) In one out of 23 *hG::Gli2N*^+^ mice, a tumor in the cerebellum (scale bar: 1 mm) was observed and is illustrated here. Besides H&E staining (Figure 3J, scale bar: 20 and 50 μm) and Ki67 staining (Fig, 3K, scale bar: 20 and 50 μm), also Nmyc staining was applied (Figure 3L, scale bar: 20 and 50 μm). (M–O) The single of the 12 transgenic *hG::NMYC*^*Fl/+*^*;Gli2N*^*+*^ mice with tumor-like structures is displayed (scale bar: 1 mm). Tumor-like structures demonstrated in the brain stem (Figure 3N) and in the subventricular zone (SVZ) (Figure 3O) are shown after H&E, Ki67, and Nmyc staining (scale bar: 20 μm H&E and 50 μm Ki67 and Nmyc). (P) Mouse body weights of *hG::Gli2N*^*+*^, *hG::NMYC*^*Fl/+*^*;Gli2N*^*+*^, *hG::p53*^*Fl/Fl*^*;Gli2N*^*+*^, and control mice (*p53*^*Fl/Fl*^*;Gli2N*^*+*^*, NMYC*^*Fl/+*^*;Gli2N*^*+*^, and *Gli2N*^*+*^) are displayed. For statistical analysis, a grouped analysis with multiple *t* test was applied compared with the sum of the control mice (*p* ∗0.03 for of *hG::NMYC*^*Fl/+*^*;Gli2N*^*+*^ mice). Data are shown as mean ± standard error of mean (SEM), and *p* values less than 0.05 were considered significant.

In *hG::p53*^*Fl/Fl*^*;Gli2N*^*+*^ animals (Figure 4A), giant cells were observed close to the piriform cortex (Figure 4B), near the hippocampus area and caudate putamen (Figure 4C), and in the cerebellum (Figure 4D). These atypical cells expressed Ki67 (Figures 4B–4D, insets). In Figure 4E, *hG::p53*^*Fl/+*^*;Gli2N*^*+*^ mice are demonstrated in a more precise analysis using two further immunohistochemically markers, i.e., Olig2 (Oligodendrocyte transcription factor 2) and Cyclin-D1.^30^^,^^31^^,^^32^^,^^33^ Remarkably, in the *hG::p53*^*Fl/+*^*;Gli2N*^*+*^ mice, we saw Olig2 expression in white matter as well as in the giant cells described earlier (Figures 4E and 4G). Besides Olig2, Cyclin-D1 expression was demonstrated in the giant cells (Figures 4F and 4H). With these observations, we have good evidence that these Ki67-positive cells do not belong to the granule cell lineage.

One out of 23 *hG::Gli2N*^*+*^mice developed a large tumor in the hindbrain (Figures 4I–4L) containing Ki67-positive proliferating cells (Figure 4K), but hardly any expression of NMYC, which is a typical marker of SHH-MB in humans (Figure 4L). Also, this tumor appears to arise from the brain stem, but not from the external granular layer of the cerebellum, where SHH-MBs usually appear. One animal out of 13 *hG::NMYC*^*Fl/+*^*;Gli2N*^*+*^ mice (Figure 4M) showed proliferative cell clusters in the ventral brain stem (Figure 4N) and in the subventricular zone (SVZ, Figure 4O). These structures show a clear Ki67, but barely NMYC staining. This mouse also showed cerebellar abnormalities causing symptoms as observed for *hG::Gli2N*^*+*^ mice. Our observations suggest that this disorder could be GLI2 dependent. However, we did not see this in mice with additional homozygous loss of *p53* or simultaneously homozygous *NMY*C expression. Table 1 demonstrates the incidence for the giant cells (60%, 7 out of 12), tumor development (4.4%, 1 out of 23), and tumor-like structures (7.7%, 1 out of 13). If considering the body weight of the mice compared to control mice (*Gli2N*^*+*^, *NMYC*^*Fl/+*^*;Gli2N*^*+*^, and *p53*^*Fl/Fl*^*;Gli2N*^*+*^ mice), it stands out that after P5, *hG::p53*^*Fl/Fl*^*;Gli2N*^*+*^, *hG::Gli2N*^*+*^, and *hG::NMYC*^*Fl/+*^*;Gli2N*^*+*^ mice have a lower body weight, which is significant in the case of *hG::NMYC*^*Fl/+*^*;Gli2N*^*+*^ (Figure 4P). These results implicate that regulation of GLI2 is essential for the cerebellar development, but a development of childhood SHH-MB either requires further genetic drivers or arises in cell types outside the *hGFAP*-positive lineage.

**Table 1:** Incidence of brain abnormalities

Genotype	Abnormalities	Incidence
*hG::p53*^*Fl/Fl*^*;Gli2N*^*+*^	Giant cells	60% (7 out of 12)
*hG:Gli2N*^*+*^	Tumor (Cerebellum)	4.4% (1 out of 23)
*hG::NMYC*^*Fl/+*^*;Gli2N*^*+*^	Tumor-like structures (Cerebrum)	7.7% (1 out of 13)

### Screening of spinal cords and specific immunohistochemically staining of hG:p53^Fl/+^; Gli2N ^+^ mice

**Figure 5:**
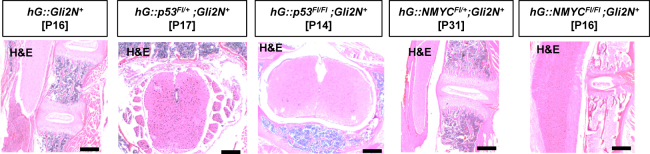
Representative H&E stains of the spinal cord Representative H&E stains of the spinal cord of all five mouse models demonstrate normal appearance and no metastasis or tumors-like structures. Spinal cords were cut longitudinal or transversal (scale bar: 200 μm).

We next investigated the spinal cords of the five different mouse models, since 2 out of 14 *hGFAP-cre::CLEG2*^*Fl/+*^mice described by Han et al. showed tumor development in the brain or spinal cord.^28^ However, H&E-stained spinal cords of our mice did not show any metastasis or tumor-like structures (Figure 5).

### Western blot analysis verifying murine GLI2, human Gli2N, Gli1, and NMYC expression in mouse cerebellum

**Figure 6:**
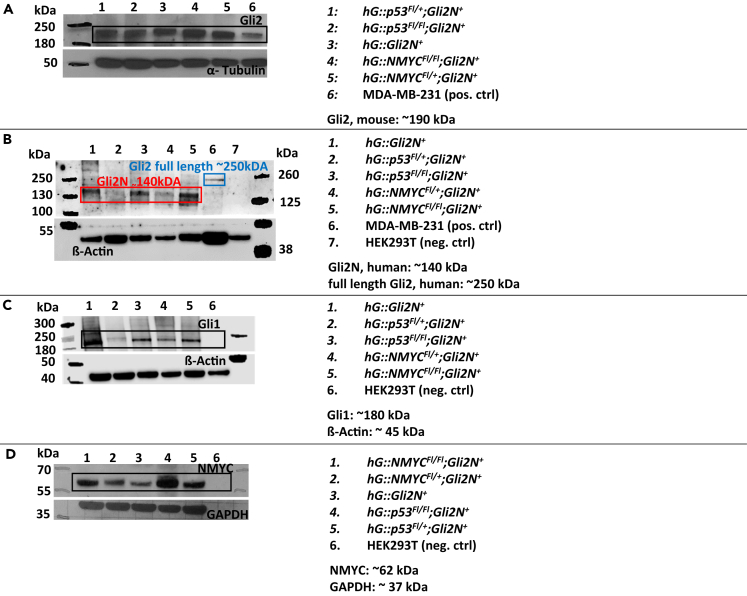
Western blot analyses (A) The lysates of fresh frozen whole cerebellum tissue were fractionated by SDS-PAGE and processed to a western blot membrane. The membrane was incubated with the GLI2-antibody PA1-28838 and the secondary antibody anti-rabbit HRP-conjugated P044801-2. α-Tubulin was used as housekeeping control. Expected band at ∼190 kDa was detected. (B) Western blot analysis using a Gli2 antibody which detects the middle region of human Gli2 and can so detect Gli2N and human full-length Gli2. The truncated Gli2N was detected in all mouse models, the human full-length Gli2 in the control (human breast cancer cell line MDA-MB-231). β-Actin was demonstrated as housekeeping gene for the analysis. (C) In this western blot analysis, Gli1 was detected in all mouse models, even though *hG::p53*^*Fl/+*^*;Gli2N*^*+*^ and *hG::NMYC*^*Fl/+*^*;Gli2N*^*+*^ show a distinct weaker band than the homozygous mouse model. (D) NMYC expression was demonstrated in all mouse models, GFAP was used as housekeeping control.

We further examined GLI2 expression in our mouse models, using western blot analysis (Figure 6). We detect a predominant band at ∼190 kDa corresponding to murine GLI2 in mouse brain verifying the expression of murine GLI2 in the transgenic mouse models (Figure 6A). The GLI2 polyclonal antibody (PA1-28838) corresponds to the amino acids 1193–1209 of mouse Gli2 with homology to human Gli2.

In Figure 6B, the expression of human Gli2N is demonstrated. The GLI2 antibody (Middle Region, ABIN2777474) recognizes the human sequence RNDVHLRTPL LKENGDSEAG TEPGGPESTE ASSTSQAVED CLHVRAIKTE from human Gli2 in the middle region, which is also found in our Gli2N construct and therefore in the Gli2N expressing mouse models. In western blot analyses, this detection antibody can verify human Gli2N isoform as well as the full-length Gli2. In Figure 6B, all five mouse models show a band at the expected size for Gli2N, albeit the expression in *hG::p53*^*Fl/+*^*;Gli2N*^*+*^ and *hG::NMYC*^*F/l+*^*;Gli2N*^*+*^ mice was weaker compared to the corresponding homozygous mouse model and the *hG::Gli2N*^*+*^ mice (Figure 6B). HEK293T cells were used as negative control and did not show any band as expected. MDA-MB-231 were used as positive control and showed, as expected, a band at the level of the full-length Gli2.

Gli1 is known to be an early target gene induced by SHH signaling, beside PTCH1 and PTCH2.^34^ To demonstrate SHH target gene activity in our mouse models, we also did a western blot analysis with a Gli1-specific antibody. All mouse models express Gli1 in the cerebellum (Figure 6C). However, as also observed in the western blot with the Gli2N antibody, the heterozygous models *hG::p53*^*Fl/+*^*;Gli2N*^*+*^ and *hG::NMYC*^*Fl/+*^*;Gli2N*^*+*^ also show weaker expression here compared to the homozygous mice and *hG::Gli2N*^*+*^ mice. To demonstrate the NMYC expression in the different mouse models, a western blot analysis using a specific NMYC antibody was done. All mouse models express NMYC (Figure 6D; NMYC) showing a clear and strong band. The constitutively expressed housekeeping genes used here were α-tubulin (Figure 6A), β-actin (Figures 6B and 6C), and GAPDH (Figure 6D).

## Discussion

Even though it is believed that GLI2 acts as an oncogenic factor in SHH-MB, it is not yet clear how *GLI2* mutations act in MB development.^7^^,^^10^ Therefore, we established the described mouse models using a dominant-active GLI2N lacking the N-terminal repressor domain of GLI2 (missing 328 amino acids).^10^ GLI2N is more accomplished than full-length GLI2 to activate cell proliferation in HH-independent means.^10^ Likewise, Han et al. used GLI2N and named it CLEG2, derived from mouse models from Pasca di Magliano et al.,^28^^,^^35^ both models using the *CLEG2* transgene with a constitutively active *CAG* promoter driving expression of EGFP in absence of Cre expression. A polyA sequence prevents the transcription of the *GLI2N* transgene, which is found at the 3-terminus of the EGFP cDNA, flanked by *loxP* sites.^35^

In our mouse model, we used the GLI2N DNA from the pCS2-MT-GLI2deltaN plasmid and cloned it into the pCAG-loxPSTOPloxP-ZsGreen vector.^10^ By this, our construct was different, and we used ZsGreen for possible tumor detection. Within these unique mouse models, the survival rate of our *hG::CAG::Gli2N*^*+*^ mice was comparable to *hGFAP-cre::CLEG2*^*fl/+*^ mice from Han et al. described previously.^28^ In our mouse model, besides Gli2, additional mutations (NMYC and p53) were chosen because of the following reasons: On the one hand, ∼10% MB cases show a GLI2 activity and 18% also show a genetic event directly targeting the abundance or rather stability of MYCN. In a cohort of SHH-MB patients, *GLI2* and *MYCN* amplifications also occurred simultaneously.^6^^,^^9^^,^^12^ On the other hand, in SHH/TP53^mt^ MB patients (generally children, 4–17 years), amplification of *GLI2*, *MYCN*, or *SHH* arises, while mutations in *SUFU* and *SMO* are rare or absent.^9^ Therefore, beside *hG::Gli2N*^*+*^mice, *hG::p53;Gli2N*^*+*^ and *hG::NMYC;Gli2N*^*+*^ (the last two homozygous or heterozygous) mice were also established. Anyhow, none of our transgenic mice developed MB. In some animals, tumors/tumor-like structures in the brain occurred, although very infrequently. This is comparable to the observations by Han et al.,^28^ where only 2 out of 14 *hGFAP-cre::CLEG2*^*Fl/+*^mice developed a tumor in the brain or spinal cord, but no MB. In our mouse model, we detected a large tumor near the brain stem, pons, and cerebellum in 1 out of 23 *hG::Gli2N*^*+*^mice, which was comparable to the one described for *hGFAP-cre::CLEG2*^*Fl/+*^ mice by Han et al.^28^ As postulated by Han et al., we agree that it is most likely not an SHH-MB. Apart from the lacking expression of MYCN, the localization of this lesion is not typical for SHH-MB, which usually origin from the external granular layer, i.e., the surface of the cerebellum. Yet, we did not find tumors in the spinal cord. This was not only true for the p53-deficient model but also for the model overexpressing MYCN. This also indicates that overexpression of MYCN in *hGFAP*-positive cells is not sufficient to drive MYCN-associated ependymoma that were recently described in humans.^36^ Additionally, we observed that in *hGFAP*-expressing cells, *MYCN* overexpression alone did not result in the development of MB. Also, mice with a single copy of MYCN as well as a combination of a Brg1 knockout and an overexpression of MYCN in multipotent neural stem cells or cerebellar granule neuron precursors were not adequate to drive brain tumor formation such as MB in mice.^33^ Ectopic cell clusters in the cerebellum detected in *hGFAP-cre::CLEG2*^*Fl/+*^mice were identified in virtually all of our 23 *hG::Gli2N*^*+*^ mice and likewise in *hG::p53*^*Fl/Fl*^*;Gli2N*^*+*^ mice.^28^ The latter showed a cerebellar disorder comparable to mice with only GLI2 overexpression.

*hG::p53*^*Fl/Fl*^*;Gli2N*^*+*^ mice showed Olig2 expression in the so-called giant cells, which were also Cyclin-D1 positive. Olig2 was identified as transcription factor for expression of myelin-associated genes in cells of the oligodendrocyte lineage.^30^ Cyclin-D1, a transcriptional target in the SHH pathway, directly regulates proliferative and immature states of cerebellar granule cell progenitors.^31^ Furthermore, Gli1 also showed strong expression in cell lysates of *hG::Gli2N*^*+*^ and *hG::p53*^*Fl/Fl*^*;Gli2N*^*+*^ mice as well as all other described mouse models. *Gli1* is a target and mediator of Shh signaling in e.g., ventral neuronal differentiation. It was initially acknowledged as an amplified nuclear oncogene in human sarcomas or gliomas.^34^ Although the SHH signaling pathway is most likely activated, a heterozygous loss of *P53* additionally to the *GLI2* overexpression did not lead to development of MB, but to neurological symptoms such as ataxia, paralysis, and bodyweight loss, also observed for *hG::Gli2N*^*+*^ mice.

60% of *hG::p53*^*Fl/Fl*^*;Gli2N*^*+*^ mice showed proliferating giant cells in the brain. Indeed, loss of TP53 is frequently observed in large cell MB as it is in so-called giant cell glioblastoma.^32^^,^^37^^,^^38^ As mentioned, the giant cells in the *hG::p53*^*Fl/Fl*^*;Gli2N*^*+*^ mice also showed positive Olig2, Cyclin-D1, and Ki67 staining, but no NeuN staining. With these observations, we have good evidence that these giant cells do not belong to the granule cell lineage. One could also postulate that these giant cells are early progenitor oligodendrocytes.

As mentioned previously, apart from models with loss of P53, we developed *hG::NMYC*^*Fl/Fl*^*;Gli2N*^*+*^ and *hG::NMYC*^*Fl/+*^*;Gli2N*^*+*^ mice. In the latter, we observed in one animal tumor-like structures that proliferate in the SVZ and brain stem as well as cerebellar disorder in 4 out of 13 mice. We observed a pathological unstructured cerebellum only in *hG::NMYC*^*Fl/+*^*;Gli2N*^*+*^ mice or in mice with a heterozygous deletion of p53. Homozygous expression of NMYC or homozygous deletion of p53 did not lead to this cerebellar structure. One could postulate here that the overexpression (Gli2N) and knockdown/loss (p53) were potentially not exactly parallel events in our models and influence each other. Nevertheless, we demonstrate Gli2N and NMYC expression in all mouse models described here, although a markedly weaker expression of Gli1 and Gli2N was detectable in *hG::p53*^*Fl/+*^*;Gli2N*^*+*^ and *hG::NMYC*^*Fl/+*^*;Gli2N*^*+*^ animals.

Since more than a quarter of MB patients show activation of the SHH signaling pathway shown by GLI2/1 immunopositivity,^11^^,^^39^ activated forms of GLI2 preserve pathway activity and support survival of the tumor cells, indicating GLI2 activity as a key driver for MB.^40^ High level of Gli2 expression has been shown in several mouse models, but as mentioned, the overexpression alone is not sufficient to drive MB development,^20^^,^^21^^,^^22^ but basal cell carcinoma (BCC) in the skin.^25^ In contrast, an orthotropic granule neuron progenitors (GNP) transplantation mouse model was shown to develop MB development after Gli1 expression. Here, primary GNPs expressing GLI1/GFP were injected and resulted in cerebellar tumor formation.^41^ Since this was not a transgenic mouse model, it is difficult to compare to our mouse models, because it is another model system. But, transgenic mice with a loss of *Sufu* alone do not show MB formation, because GLI2 activation is inadequate.^7^ However, *Sufu* loss (knockout-background) and *Spop* (E3 ubiquitin ligase) re-establishes GLI2 activation and MB is induced.^7^ Therefore, we postulated that our mouse models could be an option for a MB-mouse model, which mimics GLI2 overexpression and additional mutations in the SHH pathway, since Gli2N expression could be measured in all five mouse models, but in both heterozygous models it was weaker compared to the others.

Nevertheless, this work adds valuable information to the research for transgenic MB mouse models and origination of MB development and paves the way for further investigation.

### Limitations of the study

Limitations of the study include the following: (1) the low number of *hG::NMYC*^*Fl/Fl*^*;Gli2N*^*+*^ mice. In the matings where one expected *hG::NMYC*^*Fl/Fl*^*;Gli2N*^*+*^ mice, less pups were born compared to the births after the mating for the other described mouse models. Additionally, pre-weaning loss was a problem. Since we stick to the three Rs principle, we disclaimed unessential breeding’s to minimize an excess of mice with a wrong genotype. (2) The combination of *GLI2* and *NMYC* or G*LI*2 and *p53* was neither sufficient for the development of SHH-MB. The regulation of GLI2 is therefore crucial for proper cerebellar development. Nevertheless, these data suggest that the development of childhood SHH-MB is either occurring in cellular origins outside the hGFAP-positive lineage or needs an additional genetic driver modification. Regarding the last point, we are currently working on new models.

## STAR★Methods

### Key resources table

**Table:** 

REAGENT or RESOURCE	SOURCE	IDENTIFIER
**Antibodies**		

rabbit polyclonal anti-Ki67	Abcam	Cat# ab15580; RRID:AB_443209
rabbit monoclonal anti-Nmyc	Cell Signaling Technology	Cat# 51705; RRID:AB_2799400
Rabbit monoclonal anti-NeuN	Millipore	Cat# MAB377C3; RRID:AB_10918200
goat polyclonal anti-Olig2	R and D Systems	Cat# AF2418; RRID:AB_2157554
Rat monoclonal anti-Calbindin	Leica Biosystems	Cat# NCL-CALBINDIN; RRID:AB_563448
Rabbit monoclonal anti-Cyclin D1	Abcam	Cat# ab134175; RRID:AB_2750906
Rabbit polyclonal anti-Gli2	Thermo Fisher Scientific	Cat# PA1-28838; RRID:AB_2111904
Mouse monoclonal anti-Gli1	Santa Cruz Biotechnology	Cat# sc-515751; RRID:AB_2934097
Rabbit polyclonal anti-Gli2 (middle region)	antibodies online	Cat# ABIN2777474
Goat Anti-Rabbit Immunoglobulins/HRP antibody	Agilent	Cat# P0448; RRID:AB_2617138
Rabbit monoclonal anti-ß Actin	Cell Signaling Technology	at# 8457; RRID:AB_10950489
Mouse-monoclonal anti-alpha Tubulin	GeneTex	Cat# GTX628802; RRID:AB_2716636
Rabbit polyclonal anti- GAPDH	GeneTex	Cat# GTX100118; RRID:AB_1080976

**Bacterial and virus strains**		

NEB® 5-alpha Competent E. coli (High Efficiency)	NEB	Cat#C2987HVIAL

**Critical commercial assays**		

ultraView Universal DAB Detection Kit	Roche	05269806001
OptiView DAB Detection Kit	Roche	06396500001
SuperVision 2 HRP Kit (mouse/rabbit) Polymer-Kit	DCS	PD000KIT
Dako EnVision®+ Dual Link System-HRP (DAB+)	Dako	K4065
NEBuilder® HiFi DNA Assembly Cloning Kit	NEB	E5520S

**Experimental models: Cell lines**		

MDA-MB-231	ATCC	RRID:CVCL_0062
HEK-293T	ATCC	RRID:CVCL_0045

**Experimental models: Organisms/strains**		

*hGFAP-cre mice*	The Jackson Laborytory	RRID:IMSR_JAX #004600
*lsl-p53*^*e2-e10*^*mice*	The Jackson Laborytory	RRID:IMSR_JAX #008462
*lsl-NMYC* mice	N/A	Fielitz et al. 2016, https://doi.org/10.18632/oncotarget.12766
C57BL/6J	The Jackson Laborytory	RRID:IMSR_JAX:000664

**Oligonucleotides**		

See Table S1	metabion	N/A

**Recombinant DNA**		

pCS2-MT-GLI2deltaN plasmid	Addgene	RRID:Addgene_17649
pCAG-loxPSTOPloxP-ZsGreen	Addgene	RRID:Addgene_51269

**Software and algorithms**		

Prism 8.4.3 software	GraphPad	RRID:SCR_002798
Bio Rad ChemiDoc MP Imaging System	BioRad	RRID:SCR_019037

### Resource availability

#### Lead contact

Further information and requests for resources and reagents should be directed to and will be fulfilled by the lead contact, Prof. Dr. Ulrich Schüller (u.schueller@uke.de).

#### Materials availability

Plasmids generated in this study: To generate *CAG-lsl-Gli2* mice, the G*LI2* encoding sequence from a pCS2-MT-GLI2deltaN plasmid (#17649, Addgene, Watertown, MA, USA) was used and was cloned into a pCAG-loxPSTOPloxP-ZsGreen vector (#51269, Addgene), which is in detail described in the “STAR Methods”. The plasmids are available from the lead contact with a completed Materials Transfer Agreement.

Mouse lines generated in this study (*CAG-lsl-Gli2N*, *hGFAP-cre::CAG-lsl-Gli2N* and *hGFAP-cre::p53–CAG-lsl-Gli2N, hGFAP-cre::lsl-NMYC-CAG-lsl-Gli2N* homozygous or heterozygous) are made in-house in cooperation with the Transgenic Mouse Facility, ZMNH/UKE, Hamburg, Germany and are available upon request from the lead contact.

### Experimental model and study participant details

#### Animals

Mice of both sexes were used for the experiments and were kept in individually ventilated cages (IVC) on a constant light-dark rhythm of 12/12 hours, water and food were given *ad libitum*. The experimental procedures were performed in accordance with the German Animal Welfare Act and approved by the Government of Hamburg, Germany. Pronuclear injection was performed at the Transgenic Mouse Facility, ZMNH/UKE, Hamburg, Germany. Positive founders or heterozygous animals were selected by genotyping and identified by PCR. The founder animal was chosen for further breeding upon the following criteria: No phenotype, good breeder, no leaky expression before removal of the Stop-cassette, sufficient expression of the transgene after removal of the Stop-cassette.

Female mice were mated from at least 12 weeks (p84) of age and male mice from at least 8 weeks (p56) of age. The ages of the animals that entered the experiment are shown in the survival curves (Figure 1).

### Methods details

#### Generation of CAG-lsl-Gli2 mice

To generate *CAG-lsl-Gli2* mice, the G*LI2* encoding sequence from a pCS2-MT-GLI2deltaN plasmid (a gift from Erich Roessler (Addgene plasmid # 17649 ; http://n2t.net/addgene:17649 ; RRID:Addgene_17649, Addgene, Watertown, MA, USA)^10^ was cloned into a pCAG-loxPSTOPloxP-ZsGreen vector (a gift from Pawel Pelczar (Addgene plasmid # 51269; http://n2t.net/addgene:51269; RRID:Addgene_51269))^42^ using the NEBuilder® HiFi DNA Assembly Cloning Kit and Q5® High-Fidelity DNA Polymerase (NEB, Frankfurt, Germany). To verify the correct construct, sequencing analyses were done using specific oligonucleotides. The cre-responsive fluorescent reporter plasmid contains a STOP-cassette flanked by loxP sites followed by ZsGreen. The activity of Cre-driver candidates in mice, which is e.g. Gli2N in our model, can be detected using a ZsGreen reporter plasmid, based on the STOP cassette of the CAG-floxed ZsGreen plasmid. In principle, one could see a specific expression of ZsGreen in the appropriate tissues as prescribed by the expression pattern of Cre recombinase. So in the presence of Cre recombinase, loxP site-specific excision of the STOP cassette occurs which results in expression of the ZsGreen gene driven by the ubiquitously active chicken β-actin promoter, which is linked with a CMV early enhancer (CAG). Pronuclear injection of the SapI/NotI-fragment into C57BL/6J mice was performed according to standard procedures (Transgenic Mouse Facility, ZMNH/UKE, Hamburg, Germany). Positive founders or heterozygous animals were selected by genotyping and identified by PCR using the following primer pairs: GCCTCTGCTAACCATGTTCATGCCTTC and GATCTAGCTTGGGCTGCAGGTCGAG, CCCGCCTGGAGAACCTGAAGACA and CTCTCGGTCTTGATGGCTCTGACGT, CATCATGGATGATGGCGATCACTCGAG and CCTTGGTCAGGCCGTGCTTGGACT. PCR genotyping was performed with DreamTaq Polymerase (Thermo Fisher Scientific, Waltham, MA, USA) using standard protocols. The founder animal was chosen for further breeding upon the following criteria: No phenotype, good breeder, no leaky expression before removal of the Stop-cassette, sufficient expression of the transgene after removal of the Stop-cassette.

#### Generation of mouse models and genotyping

*hGFAP-cre* animals (JAX #004600) were bred with *CAG-lsl-Gli2N* animals. *CAG-lsl-Gli2N* mice were bred with *lsl-p53*^*e2-e10*^ mice (JAX #008462) or with *lsl-NMYC* mice.^43^ For double mutant mice, the *CAG-lsl-Gli2N* mice interbred with *p53*^*e2-e10*^ mice were crossed with *hGFAP-cre::p53* animals, resulting in *hGFAP-cre::p53–CAG-lsl-Gli2N* animals. Similarly, *lsl-NMYC::lsl-CAG-Gli2N*mice were crossed with *hGFAP-cre::lsl-NMYC* mice, resulting in *hGFAP-cre::lsl-NMYC-CAG-lsl-Gli2N* mice. Mice of both sexes were used for the experiments and were kept in individually ventilated cages (IVC) on a constant light-dark rhythm of 12/12 hours, water and food were given *ad libitum.* The experimental procedures were performed in accordance with the German Animal Welfare Act and approved by the Government of Hamburg, Germany. The termination criteria which were use in the here described mouse model were weight loss >15% over 24 h, altered grooming condition, shaggy coat, changed posture, segregation from the group lasting for more than 2 days, paralysis which means partial or complete loss of function of one or more extremities, akinesia which means lack of movement to immobility, motor disorders such as gait (ataxia), and balance disturbances. Additionally decreased motor activity (decreased reaction to touching the mouse) and indications of pain (e.g. increased respiratory rate, biting, aggressiveness, lethargy, photophobia, prolonged sleep, increased defensive reaction) are part of the stop criteria. To determine the genotype of the mice, DNA was extracted from tail tips at postnatal day 3 (P3). Genotyping PCR analysis was done with the following oligo nucleotides: Gli2 CATCATGGATGATGGCGATCACTCGAG, CATCATGGATGATGGCGATCACTCGAG and CCTTGGTCAGGCCGTGCTTGGACT, p53 GCACCTTTGATCCCAGCACATA and CACAAAAAACAGGTTAAACCCAGC, NMYC ACCACAAGGCCCTCAGTACC, TGGGACGCACAGTGATGG, CTGAGTGACAGCACCCCTTT, GTTTCCTCCGTGGTGAGGTT, CTCTTCCCTCGTGATCTGCAACTCC and CATGTCTTTAATCTACCTCGATGG.

#### Immunohistochemistry of brain and spine sections

Mouse brains and spines were fixed in 4% paraformaldehyde for at least 24 h. Spines were decalcified in Osteosoft® (MerckChemicals, Darmstadt, Germany) for at least 48 h additionally. The tissue for paraffin-embedded sections was dehydrated, embedded, and cut in 2–5 μm-thick sections. Sections were then H&E-stained or stained on a Ventana Benchmark XT System using standardized protocols titrated and optimized for each antibody ((ultraView Universal DAB Detection Kit and OptiView DAB Detection Kit (Roche)). The used antibodies were rabbit anti-Ki67 (ab15580, Abcam, Cambridge, UK, dilution 1:100), rabbit anti-Nmyc (tech517055, Cell signaling, Frankfurt, Germany, dilution 1:50), rabbit anti-NeuN (MAB 377, Sigma Aldrich, Taufkirchen, Germany, dilution 1:50), goat anti-Olig2 (AF2418, R&D Systems, Minneapolis, MN, USA, dilution 1:50) and rabbit anti-Calbindin (NCL-CALBINDIN, Novacastra/Leica Biosystems, Wetzlar, Germany, dilution 1:400). Histological photomicrographs were recorded using an Olympus B43 or a Leica M165 FC microscope. Staining with Cyclin D1 antibody (ab134175, Abcam, Cambridge, UK, dilution 1:100) was done as followed: Heat-mediated antigen retrieval at 99°C in 10 mM sodium citrate buffer (pH 6.0) for 25 min was made. Afterwards, the sections were incubated in 10% hydrogen peroxide to inactivate endogenous peroxidase activity and washed in PBS two times. Prior to the incubation with the primary antibody (Cyclin-D1) the sections were blocked for 30 min (I-Block, Life Technologies, Waltham, MA. United States). The incubation with primary antibodies took place over night followed by a washing step in PBS subjected to the staining protocol of Supervision2 + System-HRP/DAB (DCS, Hamburg, Germany and DAKO, Santa Clara, CA, USA) according to manufacturer’s recommendations.

### Preparation of mouse brain lysates and Western blot analysis

For SDS-Page and Western blot analysis, lysates were prepared as described elsewhere.^44^ In brief, the fresh frozen cerebella were used to prepare homogenates in lyses buffer (RIPA Solubilization buffer) supplemented with 10× protease inhibitor and PhosStop (Roche, Basel, Switzerland). The samples were homogenized and incubated by shaking constantly followed by centrifugation The western blot membrane (PVDF or Nitrocellulose, Millipore) was incubated overnight at 4°C with GLI2 antibody (PA1-28838, Thermo Fisher), with a Gli1 antibody (sc-515751, Santa Cruz Biotechnology, Dallas, Texas, USA, dilution 1:200), a Gli2 antibody middle region (ABIN2777474, antibodies online, Aachen, Germany, dilution 1:200) and an Nmyc antibody (tech517055, Cell signaling, Frankfurt, Germany, dilution 1:1,000) subsequently incubated for 1 h with secondary HRP-conjugated antibodies (goat-anti-rabbit, P044801-2, Agilent DAKO, Santa Clara, CA, USA, dilution 1:2000 or 1:4000), and finally incubated in Clarity Western ECL Substrate (BioRad, Hercules, CA, USA) and transferred to an X-ray film according to standard protocols or recorded on a ChemiDoc MP Imaging System (Bio-Rad, Feldkirchen, Germany). For the housekeeping genes the following antibodies were used: ß-Actin (tech8457, Cell signaling, Frankfurt, Germany, dilution 1:1000), α-Tubulin (GTX628802, Genetex, CA, USA, dilution 1:2000) and GAPDH (GTX100118, Genetex, CA, USA, dilution 1:2000). The western blot analysis were repeated. The sample-size was estimated according to the specifications of the used detection antibodies.

### Quantification and statistical analysis

Statistical analysis was performed by GraphPad Prism 8.4.3 software (Graph Pad, San Diego, CA, USA). To analyze the survival of the mice Kaplan-Meier plots were utilized (Figures 1C and 1D) a Log-rank (Mantel-Cox) test was performed to test the significance of the results. As part of the survival analysis in Kaplan-Meier plots of two data sets, the hazard ratio is reported. Descriptive statistics were used for interquartile range [IQR]. For statistical analyses of the body weight of mice, a grouped analysis with multiple *t*-test was used, compared to the body weights of control mice (Figure 4M). Data are shown as mean ± standard error of mean (SEM), and *p-*values less than 0.05 were considered significant.

## Data Availability

• All data reported in this paper will be shared by the lead contact upon request. • This paper does not report original code. • Any additional information required to reanalyze the data reported in this paper is available from the lead contact upon request. All authors have approved the experiments and all experiments conform to the relevant regulatory standards.
