# Supplementary material for: hGFAP-mediated GLI2 overexpression leads to early death and severe cerebellar malformations with rare tumor formation
Source: iScience. 2023 Jul 28;26(9):107501. doi: 10.1016/j.isci.2023.107501 (PMC10440564; doi:10.1016/j.isci.2023.107501)
Supplement: Document S1. Table S1 [file mmc1.pdf]

iScience, Volume *26*

Supplemental information

hGFAP-mediated *GLI2* overexpression leads to early death and severe cerebellar malformations with rare tumor formation

Judith Niesen, Irm Hermans-Borgmeyer, Christina Krüger, Melanie Schoof, Franziska Modemann, and Ulrich Schüller

Table S1: Oligonucleotides used for PCR (related to STAR Methods)

Oligonucleotides	SOURCE	IDENTIFIER
Gli2 CATCATGGATGATGGCGATCACTCGAG	metabion	N/A
Gli2 CCTTGGTCAGGCCGTGCTTGGACT	metabion	N/A
Gli2 CATCATGGATGATGGCGATCACTCGAG	metabion	N/A
p53 GCACCTTTGATCCCAGCACATA	metabion	N/A
P53 CACAAAAAACAGGTTAAACCCAGC	metabion	N/A
NMYC ACCACAAGGCCCTCAGTACC	metabion	N/A
NMYC CTGAGTGACAGCACCCCTTT	metabion	N/A
NMYC GTTTCCTCCGTGGTGAGGTT	metabion	N/A
NMYC TGGGACGCACAGTGATGG	metabion	N/A
NMYC CTCTTCCCTCGTGATCTGCAACTCC	metabion	N/A
NMYC CATGTCTTTAATCTACCTCGATGG	metabion	N/A
Pronucleus pair 1 GCCTCTGCTAACCATGTTCATGCCTTC and GATCTAGCTTGGGCTGCAGGTCGAG	metabion	N/A
Pronucleus pair 2 CCCGCCTGGAGAACCTGAAGACA and CTCTCGGTCTTGATGGCTCTGACGT	metabion	N/A
